# Gender and teaching experience differences in occupational stress and psychological symptoms among Chinese junior high school teachers

**DOI:** 10.3389/fpsyg.2025.1684651

**Published:** 2026-01-12

**Authors:** Huimian Bian, He Jiang

**Affiliations:** 1 Department of Psychology, Wuhan Wudong Hospital, Wuhan, China; 2 Faculty of Social Sciences and Liberal Arts, UCSI University, Kuala Lumpur, Malaysia; 3 College of Music and Dance, Guangzhou University, Guangzhou, China

**Keywords:** career stage, Chinese teachers, gender comparisons, occupational stress, psychological symptoms

## Abstract

**Background:**

Teaching is a high-stress profession globally, yet the associations of gender and teaching experience with occupational wellbeing in non-Western contexts like China remain insufficiently examined, challenging the direct applicability of Western-centric stress models.

**Objective:**

This study aimed to examine differences in occupational stress and psychological symptoms by gender and teaching experience among Chinese junior high school teachers, testing the generalizability of established stress theories within this specific socio-cultural and institutional setting.

**Methods:**

A cross-sectional survey was conducted with 397 teachers from public junior high schools in Henan Province, China. Participants completed the validated Occupational Stress Questionnaire for Teachers and the Symptom Checklist-90. Data were analyzed using analysis of variance, polynomial trend analysis, and effect size calculations.

**Results:**

No statistically or practically significant gender differences were found in any stress dimension or psychological symptom domain. In contrast, a strong, nonlinear association with teaching experience emerged. Teachers with over 30 years of experience reported significantly lower overall stress and fewer psychological symptoms compared to early- and mid-career groups, with moderate-to-large effect sizes, indicating a distinct late-career protective effect.

**Conclusion:**

The absence of gender differences challenges universal assumptions about gendered stress patterns, highlighting the critical moderating role of socio-cultural context. The pronounced protective effect of long-term tenure suggests a unique career-stage dynamic within the high-pressure Chinese educational system. These findings advocate for a decisive shift in policy and intervention focus—from gender-based to experience-tailored support systems—and underscore the urgency of addressing systemic stressors, particularly for teachers in early to mid-career stages.

## 1 Introduction

Teaching is widely recognized as a highly demanding profession, characterized by substantial workloads, complex interpersonal dynamics, and high-stakes accountability pressures (Van Droogenbroeck et al., 2014). The teaching profession is now globally recognized as facing a mental health crisis, with meta-analytic evidence confirming alarmingly high prevalence rates of burnout, anxiety, and depression among educators worldwide (Agyapong et al., 2022; Halat et al., 2023; He et al., 2025). This erosion of teacher wellbeing constitutes not only a critical public health concern but also a direct threat to educational quality and student outcomes (Arens and Morin, 2016; Maricuţoiu et al., 2023). Therefore, identifying modifiable factors that buffer against these adverse effects is a research imperative. Consequently, the prevalence of psychological symptoms such as anxiety, depression, and somatic complaints is notably high among teachers, reflecting the cumulative toll of chronic work-related stress (Bartholomew et al., 2014; Tennant, 2001). Identifying the demographic factors associated with variability in stress and wellbeing is therefore critical for developing targeted support and intervention strategies for this vital population.

Gender and teaching experience are not independent demographic correlates—both interact with cultural and systemic contexts to shape stress trajectories, making their joint examination critical for context-specific insights. Among the most frequently examined demographic correlates in the occupational stress literature are gender and teaching experience. Previous studies on gender have yielded inconsistent findings. Some report higher stress and psychological symptom levels among female teachers, often attributed to factors such as greater emotional labor and work–family conflict (Kuśnierz et al., 2022). However, these patterns may not be universal and are likely moderated by specific cultural and institutional contexts. In China, the teaching profession is characterized by a unique socio-cultural landscape. While teaching offers relative employment stability, structural gender disparities persist. For instance, female teachers often face a “glass ceiling” in promotion to senior administrative roles (e.g., principalships). They may encounter gendered expectations regarding their dedication and conduct both inside and outside the school (Yu, 2025). Simultaneously, the professional experience is shaped by a tension between traditional values—such as Confucian norms that may emphasize women’s primary role in domestic spheres—and modern expectations for equal career advancement and professional competence. This complex socio-cultural environment may attenuate or reconfigure gender-based stress patterns typically observed in Western contexts, necessitating a context-sensitive investigation.

Alongside gender, teaching experience—operationalized as years of service—represents another core demographic correlate that may shape stressor exposure and appraisal. The global literature presents a notable contradiction: while some studies indicate a decline in stress over time due to the accumulation of coping strategies, others suggest an increase due to accumulated or shifting demands (Martínez-Zaragoza et al., 2020; Admiraal et al., 2023; Veliz and Mainsbridge, 2024). This inconsistency underscores that the stress–experience trajectory is shaped by systemic features, with potential for non-monotonic patterns but is likely reconfigured by specific systemic features. In China, the high-pressure context of junior high schools, defined by the pervasive influence of the Gaokao examination pipeline and frequent, top-down educational reforms, may significantly alter this trajectory. For example, the relentless pressure for student performance and the need to continuously adapt to systemic changes could sustain high stress levels across all career stages or create unique pressures for mid-career teachers tasked with implementation. Thus, the Chinese context provides a critical setting to examine how systemic forces can modulate the well-documented yet contradictory relationship between experience and occupational wellbeing.

Given the mixed findings in the broader literature and the scarcity of large-scale, quantitative studies that simultaneously examine both gender and teaching experience among Chinese junior high school teachers, the present study seeks to address this gap. Examining these two core demographic factors in tandem is crucial, as it allows for a more integrated understanding of their respective and potentially interactive roles in teacher wellbeing. Identifying distinct stress and symptom profiles across demographic subgroups can directly inform the design of targeted mental health and workload management interventions. Furthermore, such findings contribute to a more nuanced, cross-cultural understanding of how teacher characteristics relate to occupational wellbeing.

Despite the wealth of literature on teacher stress, a significant gap remains. First, large-scale quantitative studies simultaneously examining both gender and teaching experience among Chinese junior high school teachers are scarce. Second, the inconsistent findings globally regarding both variables call for context-specific investigations, particularly in high-stakes educational systems like China’s. Third, there is a lack of understanding regarding whether the potential effects of teaching experience are linear or follow a more complex, nonlinear trajectory in such contexts. Therefore, this study seeks to fill these gaps by examining whether occupational stress and psychological symptoms vary by gender and teaching experience in a large sample of Chinese junior high school teachers. Specifically, the study addresses the following research questions:

- RQ1: Are there significant differences in occupational stress and psychological symptoms between male and female Chinese junior high school teachers?
- RQ2: How do occupational stress and psychological symptoms vary across teachers at different career stages, defined by years of teaching experience?

This study aims to: (a) examine gender differences in occupational stress and psychological symptoms among Chinese junior high school teachers; (b) explore the relationship patterns between these variables and teaching experience (career stages); and (c) provide empirical evidence for targeted teacher support policies based on the findings.

## 2 Literature review

### 2.1 Occupational stress in the teaching profession

Teaching is universally acknowledged as a high-stress occupation, characterized by a confluence of chronic and acute demands. Educators navigate not only high workloads related to lesson planning, grading, and administrative duties but also the interpersonal challenges of student misbehavior, conflicts with parents and colleagues, and often insufficient organizational support (Antoniou et al., 2020). The cumulative effect of these stressors is a heightened risk for burnout, emotional exhaustion, and a decline in both mental and physical health (Ji et al., 2021; Springer et al., 2023). While personal resilience and supportive work environments can mitigate these effects, the structural nature of these stressors underscores that teacher wellbeing is not merely an individual concern but a systemic one, with direct implications for educational quality (Table 1).

**Table 1:** Common stressors and effects in teaching.

Stressor type	Examples	Effects on teachers	Citations
Workload	Lesson planning, grading, and admin tasks	Fatigue, burnout, sleep problems	Antoniou et al. (2020), Cabaguing (2022), Redín and Erro-Garcés (2020) and Mishra et al. (2020)
Student-related	Misbehavior, absenteeism, special needs	Anxiety, frustration	Antoniou et al. (2020) and Cui (2022)
Organizational	Lack of support, resources, and low pay	Job dissatisfaction, stress	
Interpersonal	Colleague/parent conflicts, poor leadership	Emotional exhaustion, stress	Kleiousi and Retali (2023)
Emotional/Physical	Chronic stress, burnout, and health issues	Mental health decline, absenteeism	Ji et al. (2021) and Springer et al. (2023)

### 2.2 The complex role of gender and socio-cultural context

A substantial body of international literature suggests that women, particularly in human-service professions like teaching, report higher levels of perceived stress, emotional exhaustion, and psychological symptoms (Bourgeault et al., 2021; Kim et al., 2020; Pinho et al., 2023). These disparities are frequently attributed to factors such as greater emotional labor and work–family conflict (Biswas et al., 2021; Kuśnierz et al., 2022). However, this pattern is not universal, with some studies in specific professional contexts finding minimal gender differences, indicating that occupational and cultural structures are key moderators (Capecchi et al., 2024; Dartey-Baah et al., 2020). This inconsistency underscores that gender differences in occupational wellbeing are not monolithic but are profoundly shaped by socio-cultural context.

In China, the teaching profession exists at the intersection of traditional values and rapid modernization. Female teachers navigate a unique structural landscape that includes relative employment stability within the state system, a documented “glass ceiling” in career advancement, and societal norms that may simultaneously demand professional excellence and primary domestic responsibility. This complex socio-cultural matrix may neutralize, amplify, or reshape the gender effects commonly observed in Western contexts (e.g., Clarke et al., 2023; Wescott and Roberts, 2024). Crucially, emerging cross-cultural studies suggest that national-level gender equity indices can moderate such disparities in workplace wellbeing (Chawla and Sharma, 2019). Therefore, investigating gender differences within the unique configuration of China’s educational system is not merely a replication but a critical test of the boundary conditions of prevailing theories, making it an essential area for empirical investigation. Therefore, directly testing for gender differences in the Chinese educational context is not merely a replication but a necessary investigation to determine whether established Western-centric models of gendered stress apply or if a distinct, context-specific pattern prevails. The current mixed evidence and unique contextual factors create a clear empirical gap that this study aims to address. Thus, the literature presents a theoretical tension between models proposing universal gendered vulnerabilities rooted in social roles and biological factors (Hyde and Mezulis, 2020; Mollayeva et al., 2018) and those advocating for a context-dependent perspective, where socio-structural factors can attenuate or even reverse these patterns (Trucco, 2020). The Chinese context, characterized by its unique blend of state-led employment stability, pervasive exam culture, and evolving gender norms, represents a critical yet understudied boundary condition for testing these competing models. Consequently, simply assuming the transferability of Western-based gender-stress models to Chinese teachers is problematic; a direct empirical test is essential to determine which theoretical perspective holds greater explanatory power in this setting.

### 2.3 Teaching experience: contradictory trajectories and systemic modulation

The relationship between years of teaching experience and occupational wellbeing is characterized by contradictory findings, suggesting a more complex picture than a simple linear progression. One strand of research posits an adaptation model, where experience fosters mastery and improved coping, leading to declining stress over time (Hascher and Waber, 2021; Martínez-Zaragoza et al., 2020). In contrast, an accumulation model suggests that chronic exposure to systemic demands, coupled with mid- and late-career challenges, can sustain or even increase stress levels (Admiraal et al., 2023; Veliz and Mainsbridge, 2024).

This contradiction transcends a mere empirical discrepancy; it reflects a deeper theoretical question about how systemic demands interact with personal resources over time. From a Job Demands-Resources perspective, experience may primarily function as a resource that buffers stress (Gynning et al., 2025). However, in persistently high-demand environments, chronic resource depletion may occur, leading to the ‘accumulation’ pattern. The Chinese secondary education system, with its unrelenting focus on the Gaokao, epitomizes such a high-demand environment. It remains unknown whether the ‘adaptation’ or ‘accumulation’ paradigm—or perhaps a novel, threshold-based model where relief only arrives after extreme tenure—best characterizes the experience-wellbeing link in this context. Investigating this not only addresses a local gap but also contributes to refining stress and career development theories under conditions of chronic systemic pressure.

## 3 Method

### 3.1 Participants and procedure

A purposive cluster sampling strategy was employed to recruit participants. School selection was guided by explicit criteria to ensure ecological validity and a foundation of representativeness for the study’s aims. First, we focused exclusively on public junior high schools, excluding private and remote rural schools. This decision was based on two considerations: (1) Public school teachers face a more standardized and systemic set of stressors within the Chinese context (e.g., high-stakes examination pressures, non-instructional administrative burdens, and uniform performance evaluations), which aligns closely with the core objectives of this investigation into occupational stress; (2) Public schools offer greater organizational stability and standardized management, facilitating coordinated participant recruitment.

Within one prefecture-level city in Henan Province, five public junior high schools were selected from a pool of 19. The final selection ensured diversity in geographical setting (three urban and two suburban schools) and represented medium-sized institutions (each with approximately 80–100 teachers), offering a comprehensive range of subjects. The demographic profile (e.g., age, experience distribution) of the teaching staff in these schools was consistent with national characteristics for Chinese junior high school teachers, thereby enhancing the sample’s basic representativeness.

All full-time teachers (grades 7–9) with at least 1 year of experience at these schools were invited to participate. The inclusion criteria were: (1) being a full-time junior high school teacher; (2) having at least 1 year of teaching experience; and (3) providing voluntary informed consent. School administrators granted initial permission, and teachers were invited during staff meetings or via internal channels, where the study’s purpose, voluntary nature, and confidentiality were explained.

A total of 476 questionnaires were distributed to all eligible teachers across the five schools. Participants completed the questionnaires anonymously in designated settings without the presence of administrators, sealing them in provided envelopes to ensure confidentiality.

**Figure 1:**
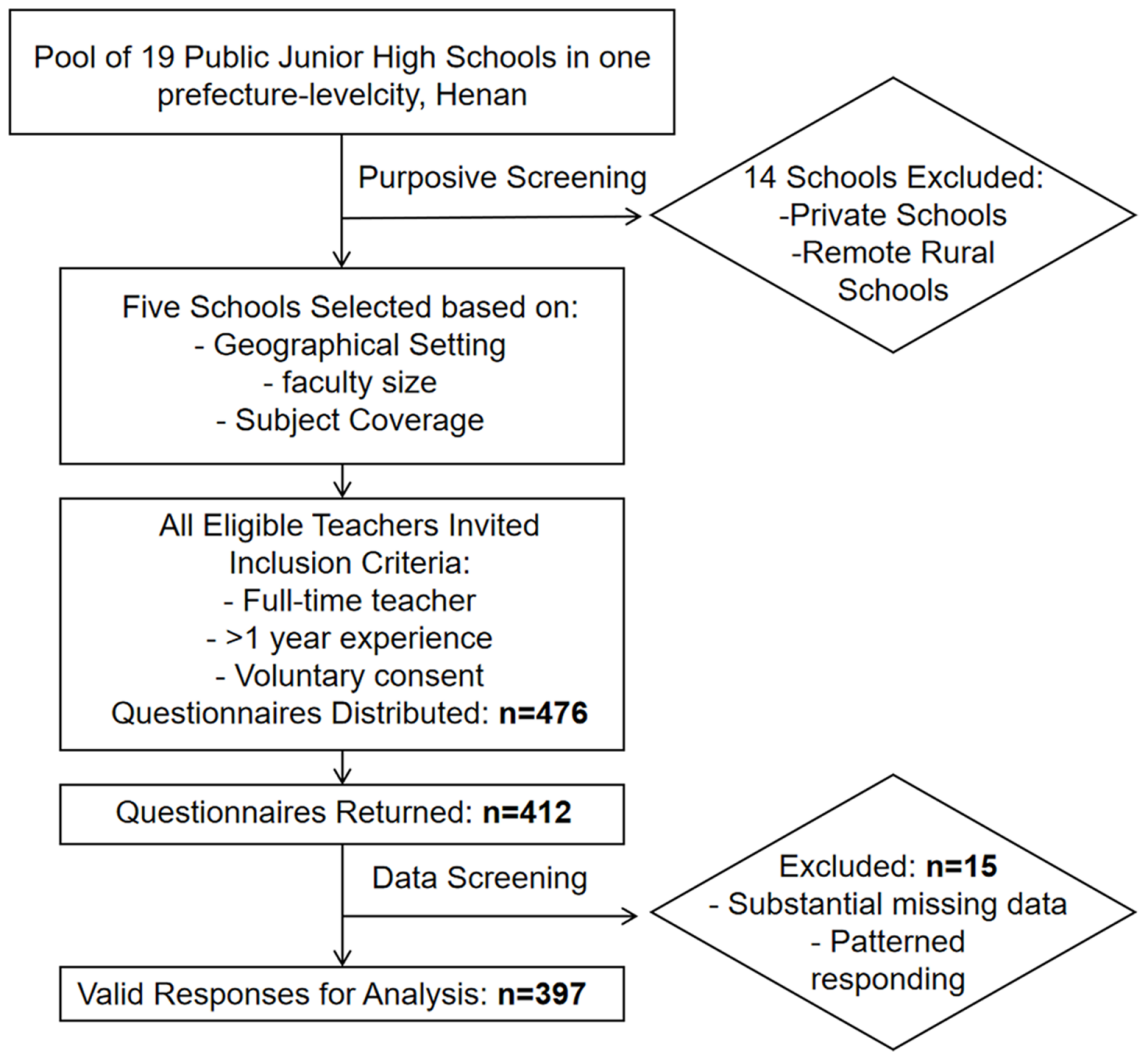
Procedure for participant recruitment.

Of the 412 questionnaires returned, 79 were excluded due to: (1) substantial missing data (≥5 key items incomplete), or (2) patterned responding (e.g., straight-lining). This resulted in 397 valid responses for baseline analysis, yielding an effective response rate of 83.4%, which meets accepted standards for social science survey research. The participant recruitment flow is summarized in Figure 1, and the demographic characteristics of the final sample are presented in Table 2.

**Table 2:** Demographic characteristics of junior high school teachers (*N* = 397).

Demographic characteristics	Categories	Frequency	%	Cumulative percentage
Gender	Male	65	16.4	16.4
	Female	332	83.6	100.0
Age (years)	20–30	75	18.9	18.9
	31–40	96	24.2	43.1
	41–50	175	44.1	87.2
	50+	51	12.8	100.0
Teaching experience (years)	Below 10	62	15.6	15.6
	11–20	110	27.7	43.3
	21–30	174	43.8	87.2
	31+	51	12.8	100.0
Degrees	Education degrees	247	62.2	62.2
	Non-Education degrees	150	37.8	100.0
Monitor	Yes	93	23.4	23.4
	No	304	76.6	100.0

As shown in Table 2, the sample comprised 397 junior high school teachers, with a significant gender imbalance favoring females (83.6%, *n* = 332) over males (16.4%, *n* = 65). In terms of age distribution, the largest proportion of participants fell within the 41–50 age group (44.1%, *n* = 175), followed by those aged 31–40 (24.2%, *n* = 96), 20–30 (18.9%, *n* = 75), and 50 years and above (12.8%, *n* = 51). Regarding teaching experience, the majority of teachers had 21–30 years of experience (43.8%, *n* = 174), while 27.7% (*n* = 110) had 11–20 years, 15.6% (*n* = 62) had less than 10 years, and 12.8% (*n* = 51) had 31 or more years. Over half of the teachers held Education degrees (62.2%, *n* = 247), whereas 37.8% (*n* = 150) did not. Additionally, a minority of the sample held Monitor roles (23.4%, *n* = 93), while most did not (76.6%, *n* = 304).

### 3.2 Measures

The study employed culturally validated psychometric instruments to operationalize the core constructs of occupational stress and psychological symptoms. As part of the initial data collection, we also administered the Mindful Attention Awareness Scale (MAAS) and the Coping Self-Efficacy Scale (CSES) to explore potential mechanistic pathways. However, as the present manuscript is narrowly focused on testing demographic differences in line with the stated research questions, these variables were not included in the current analysis.

#### 3.2.1 Occupational stress

Occupational stress was measured using the Occupational Stress Questionnaire for Teachers developed by Zhu et al. (2002). This 46-item instrument was selected for its specificity to the Chinese educational context and for evaluating six occupation-specific stress dimensions: exam pressures, student management, self-development needs, interpersonal stress, workload, and career stagnation. Items (e.g., “Students have unrealistically high expectations”) were rated on a 5-point intensity scale (0 = not at all to 4 = extremely). The original validation reported strong reliability (*α* = 0.94) and factorial validity. In the current sample, internal consistency was excellent (Cronbach’s *α* = 0.932). The Occupational Stress Questionnaire for Teachers (Zhu et al., 2002) was selected over international alternatives, such as the Teacher Stress Inventory, due to its specific relevance to the Chinese educational context. It captures culturally unique stressors related to the Gaokao examination system and centralized educational management, ensuring ecological validity for this population.

#### 3.2.2 Psychological symptoms

Psychological symptoms were measured using the Symptom Checklist-90 (SCL-90) in its Chinese adaptation (Feng and Zhang, 2001). This 90-item instrument measures mental health across nine domains (e.g., somatization, depression, anxiety). Items (e.g., “Feeling blue,” “Nervousness or shakiness inside”) are rated on a 5-point distress scale (1 = none to 5 = extreme). The scale has established norms and good psychometric properties in Chinese populations. The current study confirmed high reliability (Cronbach’s *α* = 0.957).

#### 3.2.3 Validation procedures

A pilot study (*n* = 85) was conducted to verify instrument clarity and cultural appropriateness. Cronbach’s *α* values for the main scales used in the final analysis exceeded 0.85, as shown in Table 3, surpassing conventional thresholds for group-level analyses.

**Table 3:** Reliability analysis of study instruments.

Scale	Cronbach’s *α*
Occupational stress scale	0.932
SCL-90 scale	0.957

### 3.3 Data analysis

Data were analyzed using SPSS Version 28. Descriptive statistics (means and standard deviations) were calculated separately for the gender and teaching experience groups. Independent-samples t-tests were used to examine gender differences, and one-way analyses of variance (ANOVAs) were used to assess differences across teaching experience groups. To address the shape of the trend in stress/symptoms across experience groups, polynomial contrasts were conducted following significant ANOVAs. This allowed for explicit testing of linear (straight-line), quadratic (U-shaped/inverted U-shaped), and cubic (three-directional) trends in the outcome variables. When ANOVA results were significant, *post hoc* comparisons were conducted using Tukey’s HSD test. Effect sizes were calculated using Cohen’s *d* for t-tests and partial eta-squared (*η*^2^) for ANOVAs, with thresholds of 0.20/0.50/0.80 (small/medium/large) for d and 0.01/0.06/0.14 for *η*^2^. Statistical significance was set at *p* < 0.05 (two-tailed). To address the potential interplay between our two primary demographic variables, a series of two-way ANOVAs was also conducted, with gender and teaching experience group as independent variables and each stress and symptom score as dependent variables.

### 3.4 Ethical considerations and limitations

Prior to participant recruitment, ethical approval was obtained from the ethics review board of Wudong Hospital, Wuhan, China (Approval No. 230630). The purpose, nature, and potential risks and benefits of the study were clearly explained to potential participants. Informed consent was obtained from all participants. A primary limitation of this study is the use of a convenience cluster sample from one province in China, which may restrict the generalizability of the findings to other regional and national contexts.

## 4 Results

### 4.1 Gender differences

To verify whether gender differences affect teachers’ occupational stress, Table 4 presents the ANOVA results.

**Table 4:** Descriptive statistics and gender differences in stress and symptom measures (*N* = 397).

Variable		Male (*n* = 65) M (SD)	Female (*n* = 332) M (SD)	Cohen’s *d*	*F*(1, 395)	*p*	*η* ^2^
Stress	Total stress	157.37 (24.80)	160.62 (20.92)	0.15	1.23	0.268	0.003
	Self-development stress	32.32 (7.20)	33.07 (6.46)	0.11	0.70	0.402	0.002
	Quiz/test stress	25.45 (5.74)	25.29 (4.41)	−0.03	0.06	0.807	0.000
	Workload stress	17.42 (3.83)	17.84 (3.45)	0.12	0.78	0.377	0.002
	Student-related stress	47.72 (9.31)	49.76 (8.61)	0.23	2.95	0.087	0.007
	Interpersonal stress	22.57 (4.82)	22.27 (4.55)	−0.07	0.23	0.636	0.001
	Occupational expectation stress	11.89 (2.84)	12.39 (2.67)	0.19	1.82	0.178	0.005
Symptoms	Total symptoms	214.80 (33.22)	216.65 (30.23)	0.06	0.20	0.657	0.001
	Depressive symptoms	38.72 (8.09)	39.95 (7.70)	0.16	1.37	0.243	0.003
	Anxiety symptoms	30.71 (5.53)	31.44 (5.28)	0.14	1.03	0.311	0.003
	Somatic symptoms	36.55 (6.81)	37.23 (6.34)	0.11	0.60	0.440	0.002
	Interpersonal sensitivity symptoms	29.25 (5.19)	28.89 (4.64)	−0.08	0.31	0.581	0.001

Cohen’s *d* values of 0.20, 0.50, and 0.80 are interpreted as small, medium, and large effect sizes, respectively. *η*^2^ values of 0.01, 0.06, and 0.14 indicate small, medium, and large effect sizes.

A series of independent-samples t-tests was conducted to examine gender differences in stress and psychological symptoms. Contrary to much of the international literature, no statistically significant gender differences were found for any of the outcome variables (all *p* > 0.05). As detailed in Table 4, effect sizes for all comparisons were negligible (Cohen’s |*d*| < 0.25; *η*^2^ < 0.01). For instance, for Total Stress, the difference was non-significant (*t*(395) = −1.11, *p* = 0.268, *d* = 0.15), and for Student-Related Stress, which showed the largest gender difference, the effect remained small and non-significant (*t*(395) = −1.72, *p* = 0.087, *d* = 0.23). Therefore, the findings indicate an absence of meaningful gender disparities in occupational stress and psychological symptoms within this sample of Chinese junior high school teachers.

### 4.2 Teaching experience differences

One-way ANOVAs revealed statistically significant differences across teaching experience groups for all stress and symptom measures (all *p* < 0.001; see Tables 5, 6). The effect sizes (*η*^2^) ranged from 0.052 to 0.150, indicating moderate to large practical significance.

**Table 5:** Descriptive statistics and ANOVA for teaching-experience groups on stress measures (*N* = 397).

Variable	Below 10 years (*n* = 62) M (SD)	11–20 years (*n* = 110) M (SD)	21–30 years (*n* = 174) M (SD)	31+ years (*n* = 51) M (SD)	*F*(3, 393)	*p*	*η* ^2^
Total stress	161.48 (23.78)	163.77 (17.21)	163.17 (15.02)	139.90 (33.14)	19.61	< 0.001	0.130
Self-development stress	32.48 (9.64)	33.20 (5.68)	34.53 (3.85)	27.59 (8.36)	16.50	< 0.001	0.112
Quiz/test stress	25.19 (6.80)	24.50 (3.26)	26.40 (3.64)	23.53 (6.08)	7.14	< 0.001	0.052
Workload stress	19.08 (4.01)	16.74 (3.74)	18.24 (2.82)	16.78 (3.74)	8.93	< 0.001	0.064
Student-related stress	47.61 (4.72)	53.39 (8.30)	49.65 (8.09)	42.29 (10.58)	23.04	< 0.001	0.150
Interpersonal stress	24.21 (5.10)	23.07 (3.48)	22.09 (4.29)	19.22 (5.39)	13.62	< 0.001	0.094
Occupational expectation stress	12.90 (2.69)	12.87 (2.70)	12.26 (2.18)	10.49 (3.49)	11.12	< 0.001	0.078
Pairwise comparisons (Cohen’s *d* vs. 31+ years group)							
Variable		Below 10 vs. 31+		11–20 vs. 31+		21–30 vs. 31+	
Total stress		0.66		0.67		1.13	
Self-development stress		0.60		0.68		1.33	
Quiz/test stress		0.11		0.02		0.67	
Workload stress		0.29		−0.01		0.48	
Student-related stress		0.56		0.72		0.84	
Interpersonal stress		0.82		0.60		0.63	
Occupational expectation stress		0.71		0.46		0.70	

*F*-statistics have df = (3, 393). *η*^2^ = eta squared. Cohen’s *d* was computed as (M<sub>group</sub> − M<sub>31+</sub>)/pooled SD; positive values indicate the named group has a higher mean than the 31+ years group.

**Table 6:** Descriptive statistics and ANOVA for teaching-experience groups on symptom measures (*N* = 397).

Variable	Below 10 years (*n* = 62) M (SD)	11–20 years (*n* = 110) M (SD)	21–30 years (*n* = 174) M (SD)	31+ years (*n* = 51) M (SD)	*F*(3, 393)	*p*	*η* ^2^
Total symptoms	235.47 (42.19)	215.08 (27.85)	217.67 (16.60)	191.33 (39.35)	22.67	< 0.001	0.148
Depressive symptoms	43.02 (9.94)	38.95 (7.73)	40.95 (4.59)	33.43 (9.71)	18.92	< 0.001	0.126
Anxiety symptoms	33.56 (6.56)	30.52 (4.58)	32.35 (3.53)	26.80 (7.14)	21.91	< 0.001	0.143
Somatic symptoms	39.68 (8.02)	36.45 (6.08)	38.28 (4.05)	31.49 (7.97)	21.61	< 0.001	0.142
Interpersonal sensitivity symptoms	29.89 (5.50)	28.76 (4.49)	29.64 (3.59)	25.84 (6.26)	10.10	< 0.001	0.072
Pairwise comparisons (Cohen’s *d* vs. 31+ years group)							
Variable		Below 10 vs. 31+		11–20 vs. 31+		21–30 vs. 31+	
Total symptoms		0.61		0.62		1.11	
Depressive symptoms		0.92		0.57		1.23	
Anxiety symptoms		0.96		0.58		1.21	
Somatic symptoms		1.04		0.63		1.31	
Interpersonal sensitivity symptoms		0.69		0.57		0.88	

*F*-statistics have df = (3, 393). *η*^2^ = eta squared. Cohen’s *d* was computed as (M<sub>group</sub> − M<sub>31+</sub>)/pooled SD; positive values indicate the named group has a higher mean than the 31+ years group.

The results demonstrated a clear but nonlinear pattern of occupational stress and psychological symptoms across teaching experience groups (Tables 5, 6). For most outcome variables, stress/symptom levels did not decrease steadily with experience: (1) Teachers with 11–20 years of experience reported higher Total Stress (*M* = 163.77, SD = 17.21) than those with <10 years (*M* = 161.48, SD = 23.78), and similar levels to those with 21–30 years (*M* = 163.17, SD = 15.02); (2) Only the 31+ years group showed a sharp reduction in stress/symptoms, with large effect sizes relative to all other groups (*d* = 0.66–1.13). (3) Polynomial contrasts confirmed that linear trends were not significant for most variables, while quadratic trends (indicating mid-career rebound) were significant (e.g., Total Stress: *F*(1, 393) = 18.45, *p* < 0.001). This confirms the nonlinear nature of the experience-stress relationship: stress levels remain elevated in early-to-mid career (≤30 years) and only decline sharply in the most senior group (31+ years).

It is important to note that the very large effect sizes observed in the 31+ years group may be partly influenced by its relatively smaller sample size (*n* = 51) and the use of raw scale totals rather than mean item scores. Furthermore, the cross-sectional design precludes causal inference; the lower stress among the most experienced teachers could reflect developmental adaptation, selection effects, or cohort differences in initial training.

### 4.3 Analysis of gender and teaching experience interaction effects

To explore whether the relationship between teaching experience and wellbeing outcomes differed by gender, a series of 2 (Gender) × 4 (Teaching Experience Group) between-subjects ANOVAs was conducted. The results revealed no significant interaction effects for any of the outcome variables, including Total Stress, *F*(3, 389) = 0.52, *p* = 0.670, *η*^2^ = 0.004, Total Symptoms, *F*(3, 389) = 0.48, *p* = 0.696, *η*^2^ = 0.004, or any of the subscales (all *p* > 0.05). This indicates that the trajectories of occupational stress and psychological symptoms across different career stages were consistent for both male and female teachers in this sample. No corrections for multiple comparisons were applied to these analyses in order to avoid an undue increase in Type II error, given the exploratory nature of the interaction tests; however, this increases the risk of Type I error, and the results should be interpreted with this in mind. No corrections for multiple comparisons were applied to maintain statistical power and minimize Type II errors, as recommended when testing *a priori* hypotheses (Doroudgar et al., 2022). However, readers should interpret the results with the understanding that this increases the family-wise error rate.

## 5 Discussion

This study investigated demographic correlates of occupational wellbeing among Chinese junior high school teachers. Two central findings emerged. First, we found no statistically significant gender differences in occupational stress or psychological symptoms, with effect sizes indicating negligible practical significance. Second, we identified a strong but nonlinear association between teaching experience and wellbeing. Teachers with over 31 years of experience reported substantially lower stress and fewer symptoms than their less-experienced colleagues, with moderate-to-large effect sizes. The most pronounced differences were observed between the 21–30 years and the 31+ years groups, suggesting a potential threshold effect rather than a simple linear decline.

Our null finding regarding gender differences stands in contrast to a significant body of international literature, which frequently reports higher stress and psychological symptoms among female teachers and other professionals (Bourgeault et al., 2021; Pinho et al., 2023; Kim et al., 2020). However, our results align with a smaller number of studies suggesting that gender differences are not universal and can be attenuated by specific occupational and cultural contexts (Capecchi et al., 2024; Dartey-Baah et al., 2020). This establishes an important boundary condition for theories of gendered occupational stress and underscores the profound moderating role of socio-cultural context. The absence of a mean-level gender difference in our Chinese sample does not necessarily imply equality of experience; it is plausible that male and female teachers face qualitatively different stressors that, when combined, cancel each other out in our measures. For instance, while female teachers may face a higher burden of emotional labor (Kuśnierz et al., 2022), male teachers may face heightened performance pressures in classroom management and discipline within a female-dominated profession. This dynamic is less commonly reported in Western contexts.

The robust protective association of long-term teaching experience aligns with one strand of the literature suggesting that experience can foster mastery, improved coping strategies, and professional efficacy (Hascher and Waber, 2021; Zhou et al., 2024). However, our findings of a nonlinear trajectory—with stress stable in early-to-mid career and declining only in late career—diverge from linear models of adaptation or accumulation (Admiraal et al., 2023; Veliz and Mainsbridge, 2024). This divergence highlights the critical role of systemic context. The Chinese educational environment, with its intense focus on the Gaokao examination pipeline, may create a unique stress trajectory. The ‘relief’ observed in the most senior teachers could reflect a combination of role adjustment, institutional mastery, and crucially, the accumulation of personal and social resources that buffer chronic stressors. Analogous to how formal support systems like clinical supervision can mitigate the impact of emotional demands in helping professions (Geisler et al., 2024), the accrued experience, respect, and informal support networks of veteran teachers in China may serve as a powerful protective resource against systemic pressures. This perspective aligns with the Job Demands-Resources model, where long tenure may enhance resources.

The findings strongly advocate for career-stage-specific support systems. Early-career teachers, who face significant adaptation challenges (Admiraal et al., 2023), would benefit from comprehensive mentoring and stress management training. Mid-career teachers require interventions to prevent burnout associated with cumulative demands. Critically, interventions must extend beyond the individual to address the systemic sources of teacher stress cataloged in our literature review (Antoniou et al., 2020). Policymakers and school administrators should consider systems-level changes, such as: (1) Implementing fair and transparent systems for administrative task allocation. (2) Ensuring that support programs are designed to be inclusive and accessible to teachers of all genders, acknowledging that male teachers may be equally vulnerable.

Several limitations of the present study should be acknowledged. First, the cross-sectional study design precludes causal inferences between the variables under investigation. Second, the use of a convenience sample drawn from a single Chinese province limits the generalizability of the findings to broader populations. Third, reliance on self-report measures raises the potential risk of common-method bias, which may affect the validity of the results. Fourth, while the grouping of teaching experience was determined pragmatically for analytical purposes, this approach may obscure more complex, non-linear developmental trajectories in the variable. Fifth, the notably large effect sizes observed in the 31+ years teaching experience group call for caution in interpretation; these effects may be influenced by two factors: the smaller sample size of this group and the use of raw scale totals in the analysis. Finally, given that the sample is predominantly female, it may be considered unbalanced for gender-based analyses, which could restrict the applicability of gender-related conclusions. Future studies should adopt longitudinal designs to disentangle developmental adaptation from selection effects. Furthermore, to understand the psychological process underlying the stress differentials across career stages, investigating potential cognitive mediators such as affective work rumination—a known mechanism linking job demands to exhaustion (Geisler et al., 2023)—would be valuable. Incorporating such objective or process-focused measures would complement self-report data and reveal how stressors translate into symptoms for teachers at different career points.

## 6 Conclusion

This study demonstrates that within the specific context of Chinese junior high schools, teaching experience—not gender—is the primary demographic differentiator of occupational stress and psychological symptoms. By revealing a clear absence of gender disparities—contrasting with much of the international literature—and a pronounced protective effect of long-term tenure, our findings challenge simplistic generalizations and highlight the critical importance of cultural and systemic context. This study contributes to the literature by identifying context-specific patterns, thereby challenging universalist assumptions about teacher stress and necessitating a rethinking of intervention paradigms. Ultimately, enhancing teacher wellbeing requires a dual focus: providing tailored, stage-sensitive individual supports while simultaneously addressing the overarching institutional and policy-level drivers of stress, such as the high-stakes testing culture, that shape the professional landscape for all educators.

## Data Availability

Data supporting the findings of this study are openly available in Figshare at https://doi.org/10.6084/m9.figshare.30837092.
